# Transient colonization with *Blastocystis* spp. after transmission via fecal microbiota transplantation

**DOI:** 10.1007/s10096-025-05124-6

**Published:** 2025-04-21

**Authors:** M. V. Bénard, C.M.A. de Bruijn, S. Matamoros, E.M.S. Wentink-Bonnema, M. A. Benninga, C. Y. Ponsioen, R. Zonneveld

**Affiliations:** 1 Department of Gastroenterology and Hepatology, Amsterdam Gastroenterology Endocrinology Metabolism (AGEM), Amsterdam UMC, University of Amsterdam, Amsterdam, The Netherlands; 2 Inflammatory Bowel Disease Center, Amsterdam University Medical Center, Amsterdam, The Netherlands; 3 Emma Children’s Hospital, Pediatric Gastroenterology, Hepatology and Nutrition, Amsterdam University Medical Center, University of Amsterdam, Amsterdam, The Netherlands; 4 Amsterdam Reproduction & Development Research Institute, Amsterdam University Medical Center, Emma Children’s Hospital, Amsterdam, The Netherlands; 5 Department of Medical Microbiology and Infection Prevention, Section Clinical Parasitology, Academic Medical Center, Amsterdam University Medical Center, University of Amsterdam, Meibergdreef 9, Amsterdam, 1105 AZ The Netherlands

## Abstract

**Background:**

The pathogenicity of *Blastocystis* spp. is still debated. Guidelines for feces donor screening differ in their advice to screen for *Blastocystis* spp., but when tested, its presence is a common reason for exclusion. *Blastocystis* spp. are correlated to increased bacterial alpha-diversity and distinct bacterial groups and therefore its presence may indicate favorable efficacy of fecal microbiota transplantation (FMT). The latest European consensus report no longer advices rejecting feces donors testing positive for *Blastocystis* spp. Only one paper has been published on human transmission of *Blastocysti*s spp. via frozen FMT.

**Objective:**

To investigate the transmission and long-term effects of *Blastocystis*-positive FMT, prepared with fresh (i.e., unfrozen) feces.

**Methods:**

In a trial (NCT03074227) on FMT for refractory Irritable Bowel Syndrome (IBS), adolescents (age 16–20 years) received two administrations - at baseline and after 6 weeks - of fresh allogeneic FMT from a *Blastocystis*-positive donor via nasoduodenal tube. The follow-up was 48 weeks. *Blastocystis* spp. presence, viability and subtyping were determined using microscopy, culture, PCR and sequencing.

**Results:**

Three recipients received FMT from one donor colonized with *Blastocystis* subtype 3 (ST3). At baseline, two recipients were negative for *Blastocysti*s spp. and one recipient carried ST2. Culturing revealed viable *Blastocystis* spp. in fresh donor feces but not in frozen samples. After FMT with fresh feces, the two prior-negative recipients tested positive for the donor’s ST3 at 12 weeks, but had lost this subtype by week 24 and 48. The recipient initially colonized with ST2 remained colonized with ST2 and did not acquire ST3. Transient adverse events occurred, but did not differ from patients treated with *Blastocystis*-negative FMT. No FMT-related serious adverse events emerged.

**Conclusion:**

We present the first long-term data on viable *Blastocystis* spp. transmission via fresh FMT in three cases. Transient colonization with *Blastocystis* spp. was observed, without serious FMT-related adverse events.

**Supplementary Information:**

The online version contains supplementary material available at 10.1007/s10096-025-05124-6.

## Introduction

Fecal microbiota transplantation (FMT) is highly effective for treating recurrent infections with *Clostridioides difficile* (CDI) [1, 2]. In the last decades this therapy has been a popular subject of study for many other indications [3], including Inflammatory Bowel Diseases (IBD) and Irritable Bowel Syndrome (IBS). The selection of safe and effective feces donors for FMT lies at the basis of this relatively new treatment and is directed by screening guidelines. These are based on varying quality of evidence and often designed by working groups that consist of FMT experts. In practice, the selection of safe feces donors is challenging, with only 5–25% eligibility of screened individuals in European cohorts [4]. A common reason of exclusion is the presence of eukaryote *Blastocystis* spp., accounting for up to 40% of stool screen failures [4–6]. 

*Blastocystis* spp. is a large genus of at least 38 genetically diverse subtypes [2] (ST) of which 14 have been described to occur in humans so far [7]. Of those, ST3 is the most frequently reported in epidemiological studies [8]. The pathogenic potential of *Blastocystis* spp. is unclear and may be dependent on the subtype. Although its presence, in particular ST7 [9, 10], has been associated with gastrointestinal symptoms including diarrhea [9], the prevalence of *Blastocystis* spp. is common in asymptomatic individuals, and higher than in patients with IBD [11–13], suggesting at least the possibility of a harmless carrier state.

In some prominent guidelines, including an European consensus report [14], it is advised to screen for *Blastocystis* spp. and to exclude potential feces donors upon its presence. However, accumulating evidence shows that the presence of *Blastocystis* spp. in stool is correlated with distinct features, such as an increased bacterial alpha-diversity [11, 13, 15, 16], that has been associated with FMT success [17, 18]. Some studies have identified specific subtypes, such as ST1 - ST4 [11, 15], that are linked to this increased diversity, while ST7 has been associated with lower diversity [19]. Therefore, selecting against specific *Blastocystis* spp. may also hinder the selection of (more) effective feces donors.

Only one paper has been published that describes the transmission of *Blastocystis spp.* by FMT in humans [20]. The Dutch Donor Feces Bank (NDFB) discovered to have unintentionally treated 31 patients with recurrent CDI using *Blastocystis* spp. containing donor stool, which had no effect on either efficacy or safety of FMT. Post-FMT samples were available from 16 patients, who had no *Blastocystis* spp. prior FMT, of which half tested positive for *Blastocystis* ST1 or ST3 after FMT, suggesting transmission. These results imply colonization with *Blastocystis* spp., without consequences. Subsequently, in the latest United European Gastroenterology (UEG) working consensus on FMT, *Blastocystis* spp. was no longer advised as an exclusion criterion for feces donors [21]. However, only one feces sample was tested and follow-up was short. In addition, the lack of symptoms [20] may be a result of the diminished viability of *Blastocystis* spp. after a freeze-thaw cycle used for administration of frozen FMT [22]. Therefore, more knowledge on the long-term effects of transferring viable *Blastocystis*-positive material via FMT is needed. Here, we report on three IBS patients that received fresh feces FMTs from one *Blastocystis* spp. positive donor in a Dutch randomized controlled trial [23]. 

## Materials and methods

### Characteristics of donor and patients at baseline before FMT

The FMT donor and three patients were included in the ‘Feasibility and efficacy of faecal microbiota transplantation in Adolescents with refractory Irritable bowel Syndrome’ (FAIS) trial. The FAIS trial is a randomized, double-blind, placebo-controlled trial in which patients with refractory IBS are randomized in a 1:1 ratio between donor FMT and autologous FMT. The full study protocol was previously published [23]. In this randomized controlled trial (RCT) *Blastocystis*-positive donors were not excluded which provided a post-hoc opportunity to investigate the effects of potential transmission using fresh FMT in a supervised research setting with follow-up until 1 year. The healthy donor underwent screening over a timespan of two years and tested positive with microscopy and PCR for *Blastocystis* spp. consistently (Supplementary Table 3). Microscopy on samples from that period also revealed varying presence of non-pathogenic protozoans *Entamoeba coli*, *Endolimax nana*, *Entamoeba dispar* and/or *Entamoeba hartmanni*. The three patients, adolescents aged 16–20 years, had refractory Irritable Bowel Syndrome (IBS) that did not respond to previous treatments. They scored average daily pain rates ≥ 30 mm on the pain component scale of the Irritable Bowel Syndrome Severity Scoring System (IBS-SSS) [24]. 

### Stool sampling from the donor and the patients

Fresh feces samples from the donor and patients were collected during screening at -2 weeks, and at the day of treatment at week 0 (baseline) and week 6, and were cryopreserved at − 80 °C for further molecular diagnostics. In addition, fresh feces was collected in tubes containing Sodium Acetate Formaldehyde (SAF) fixative for direct microscopy. During the screening phase the donor was tested for *Blastocystis* spp. with microscopy and PCR and tested positive with both methods. No *Blastocystis* spp. subtyping was performed during the screening- and treatment phase. Feces samples collected from the patients were analyzed for presence of *Blastocystis* spp. with microscopy and PCR during the screening phase, but not standard after the FMTs, in accordance to the RCT protocol. Further unfixed feces samples from the patients were collected and cryopreserved at − 80 °C at week 12, 24 and 48. An overview of the sampling is provided in Supplementary Table 1.

### Fecal microbiota transplantation

FMTs were prepared by mixing fresh feces from the donor with saline (0.9% NaCl) whereafter the solution was filtered through double layers of gauze. Patients received a 200 mL allogeneic FMT from the donor at baseline and after 6 weeks. The FMT was administered to the patients within 6 h after collection of feces from the donor. The infusion was delivered via a nasoduodenal tube in the duodenum, following bowel lavage with macrogol electrolytes via the same tube.

### Microscopy and cultivation of *Blastocystis* spp

For microscopy, feces samples were stained with Kop-Color stain (Elitech, Puteaux). Conventional light microscopy was performed to determine the presence and load of *Blastocystis* spp, with loads categorized by experienced technicians as ‘sporadic’, ‘few’, ‘some’, ‘moderate’, ‘many’.

To determine the viability of *Blastocystis* spp. in fresh versus frozen donor feces, we cultured samples confirmed to contain *Blastocystis* via microscopy and PCR. The culturing method was based on Tan et al. [25, 26] Conventional light microscopic examination was conducted to check for growth of *Blastocystis* spp. If no growth was observed, samples were transferred to fresh culture tubes with new liquid media and re-evaluated weekly. This process was continued for four weeks until either growth was detected or the cultures were deemed negative. From each incubation, 500 uL of material was separated and stored at -80 °C for later *Blastocystis* spp. qPCR. Five conditions were prepared: fresh unprocessed feces (A), fresh FMT (B), frozen unprocessed feces (C), frozen unprocessed feces with glycerol (D), frozen FMT with glycerol (E), and The ‘fresh’ conditions (A and B) were incubated on the day of stool collection. The frozen conditions (C, D and E) were stored at -80 °C for one week prior to incubation. The FMT conditions were prepared by mixing feces with saline in a 1:2 ratio, followed by filtration. Glycerol was added to the frozen FMT condition (E) to a final concentration of 10% as a cryoprotectant, consistent with standard practices.

### *Blastocystis spp*. identification and subtyping with molecular diagnostics

To validate the use of PCRs for detecting *Blastocystis spp.* on deep frozen feces samples, we first analyzed samples with known *Blastocystis* spp. status (positive or negative controls, including microscopic load) as assessed previously with microscopy on corresponding fresh feces samples. Samples were obtained from our donor screening cohort and patients from the FAIS and TURN2 trial (trial assessing the efficacy of anaerobic prepared FMT in adults with active ulcerative colitis, NL7770). The validation step was successful, with 10 out of 12 positive tests on microscopically confirmed *Blastocystis*-positive material, and 10 out of 10 negative PCRs on *Blastocystis*-negative material (details in Supplementary Table 2).

Frozen feces samples stored at -80 °C were thawed for 20 min before processing for *Blastocystis* SSU-rDNA detection via qPCR. Samples were divided in three aliquots, of which two contained a Stool Transport and Recovery (S.T.A.R.) Buffer (Roche, Basel). The samples in S.T.A.R. buffer were centrifuged for homogenization. To confirm the presence of *Blastocystis* DNA in the feces samples, qPCR targeting a 363 bp region of the small unit ribosomal RNA gene (SSU-rDNA; see primers in Supplementary Table 3) was performed as described earlier [27, 28]. Positive samples were then used as template for the sequencing reaction (BigDye™ Terminator chemistry; Applied Biosystems) targeting the same region of the SSU-rDNA gene (primers Blasto_seq_F and Blasto_seq_R; see Supplementary Table 3). The nucleotide sequence was determined using the 3730XL DNA Analyzer (Applied Biosystems). Sequences were processed, aligned and visualized using Geneious Prime (Dotmatics, Boston, USA). *Blastocystis* subtypes were determined by comparing sequences to the PubMLST database [29]. 

### Ethics approval

This study, being a sub-analysis from the FAIS trial, was approved by the Medical Ethics Research Committee of Amsterdam UMC (Code 018) in the Netherlands. Written informed consent was obtained from all the participants.

## Results

### *Blastocystis* spp. and other protozoa in the donor

One healthy donor that was consistently colonized with *Blastocystis* spp. (Supplementary Table 1) was included in this study. PCR and Sanger sequencing showed that this donor was colonized with *Blastocystis* ST3. Of the six donor samples collected at the day of donation for FMT, five returned indeed positive for *Blastocystis* ST3. The remaining sample also showed a positive Blastocystis PCR, but the subtype could not be determined. In addition to *Blastocystis* spp., *Entamoeba coli* and *Endolimax nana* were frequently detected in the majority of tests of the donor’s samples over a two-year period, with occasional presence of *E. histolytica/dispar* and *E. hartmanni* cysts (Supplementary Table 4).

Growth of *Blastocystis* spp. was observed in all the conditions involving fresh (i.e. non-frozen) feces, both in fresh unprocessed samples and prepared fresh FMT (Table 1). In contrast, all conditions that underwent a freeze-thaw cycle tested negative for *Blastocystis* spp., suggesting that the viability of *Blastocystis* spp. was compromised regardless of processing or the addition of glycerol.

**Table 1:**
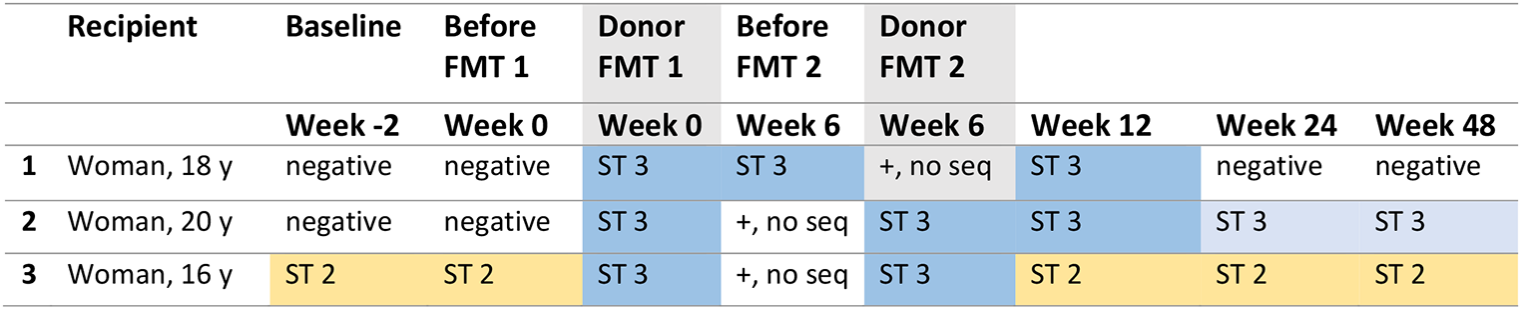
Detection of *Blastocystis* spp. After culturing samples derived from donor feces with confirmed *Blastocystis* presence, prepared under various conditions. Fecal sample 1 and sample 2 were collected on two consecutive days from the same donor. Semiquantitative microscopy evaluation: ++, many *Blastocystis* spp., +, few *Blastocystis* spp

### Transmission of *Blastocystis* spp. and other protozoa after FMT

Three female adolescents with refractory IBS (age 16–20 years) received FMT twice from this particular *Blastocystis-*positive donor (Table 2). Recipient 1 was *Blastocystis*-negative at baseline, received FMT with *Blastocystis* ST3, whereafter she tested positive for *Blastocystis* ST3 at weeks 6 and 12. During later follow-up visits she tested negative again without having received eradication treatment, suggesting spontaneous resolution. Similarly, recipient 2 acquired *Blastocystis* ST3 with a similar sequence order as from the donor, and tested positive until week 12. However, at follow-up visits, she tested positive for *Blastocystis* ST3 with a different sequence order than that originated from the donor. In contrast, recipient 3 was already colonized with *Blastocystis* ST2 at baseline, received ST3-postive donor material, but remained positive only for ST2 up to the last follow-up visit. Regarding co-occurring protozoa in the donor, microscopy revealed *Entamoeba coli* and *Endolimax nana* only in recipient 1 following FMT. Recipient 2 tested positive for Dientamoeba fragilis at baseline and remained positive after FMT, while recipient 3 did not undergo elaborate parasitology testing after FMT.

**Table 2:** Sanger PCR sequencing results of *Blastocystis* determination in frozen feces samples from three adolescents with refractory IBS and one healthy donor. Samples were collected before, during and after the FMT treatment schedule with follow-up unto week 48. Light blue cells indicate a different sequence order of *Blastocystis* ST3 than the *Blastocystis* ST-3 indicated in the dark blue cells. No sequence means that the PCR was positive for *Blastocystis* spp. But the subtype could not be determined. Abbreviations; ST; subtype, seq; sequence, y; years

Code	Condition	Microscopic evaluation sample 1	Microscopic evaluation sample 2
A	Fresh unprocessed	Positive ++	Positive ++
B	Fresh FMT	Positive ++	Positive +
C	Frozen unprocessed	Negative	Negative
D	Frozen unprocessed with glycerol	Negative	Negative
E	Frozen FMT with glycerol	Negative	Negative

### Clinical course after FMT

Recipient 1 demonstrated treatment success at both week 24 and week 48, meeting the criterion of > 50% reduction on abdominal pain intensity and frequency as assessed with the IBS-SSS, compared to baseline. The other two recipients were non-responders. Transient adverse events (AEs) including abdominal cramp, diarrhoea, and nausea, were observed in these participants, but resolved within 3 days following FMT. These AEs were comparable with the rest of the cohort and in line with expected outcomes after FMT [30]. No serious adverse events (SAEs) occurred that related to the treatment.

## Discussion

In this study, we report on three cases demonstrating that *Blastocystis* spp. transfer can occur via fresh FMT from a colonized donor. This transmission was transient and did not result in adverse events. These findings support reconsidering *Blastocystis* spp. as an exclusion criterium for FMT donors, as suggested in the latest European consensus rapport [21]. 

We show long-term results after treating adolescents with refractory IBS with fresh FMT containing *Blastocystis* spp. Unlike frozen FMT, fresh FMT does not undergo a freeze-thaw cycle, which decreases viable *Blastocystis* spp. load, as shown recently [22] and confirmed by our culture experiment. This reduced viability may have impacted previous published results on human transmission via frozen FMT [20]. Additionally, we enclosed results of *Blastocystis*-positive FMTs containing low to high amounts. Although limited by small sample size, our data are reassuring and in line with hitherto the only available paper on this subject showing that *Blastocystis*-containing frozen FMT for the treatment of recurrent CDI had no short-term effect on efficacy or safety [20]. 

Reported success rates of FMT in IBS vary widely, but recent meta-analyses of FMT in adults with IBS suggest a moderate short-term response, with significant improvements in IBS-SSS compared to placebo [31], especially with invasive administration routes [31, 32]. Further studies with larger sample sizes are needed to evaluate the comparative efficacy of *Blastocystis*-positive versus *Blastocystis*-negative FMT, in a broader range of indications beyond IBS, as well as the safety profile and the potential role of *Blastocystis* subtypes in influencing outcomes.

Investigation of the transmission of *Blastocystis* spp. via FMT comes with limitations. Of the three prior *Blastocystis* ST3-negative adolescents in this study, two tested *Blastocystis* ST3 positive post-treatment with donor FMT containing *Blastocystis* ST3. While it is likely that this ST3 infection with similar sequences are indeed originating from the donor, we can not entirely rule out the possibility that this newly acquired infection comes from another source. Even whole genome sequencing, which is not yet established for *Blastocystis* subtyping, would not resolve this uncertainty. Moreover, a few published case series showed that there is a marked day-to-day fluctuation in the shedding of these protozoa once an individual is infested [33, 34]. As a result, it is possible we have encountered false-negative results. However, due to our extended follow up and multiple testing, we believe that the chance this biased our results is low.

The lack of negative impacts of *Blastocystis* spp. transmission may point to the notion that *Blastocystis* spp. are non-pathogenic, a topic still debated in the field. Regarding IBS, some studies report an association with *Blastocystis* spp [35, 36]., though findings are inconsistent [37]. To the best of our knowledge, a causative relationship between *Blastocystis* spp. and diseases has never been demonstrated. In line with this, *Blastocystis* spp. appear more frequently in healthy asymptomatic cohorts than in symptomatic individuals [11, 12, 38, 39], suggesting at least the possibility of commensalism. Interestingly, research increasingly shows that *Blastocystis*-containing microbiota is associated to distinct microbial patterns, such as a higher bacterial alpha-diversity [11, 13, 15, 16], which is related to FMT response [17, 18]. These correlations of *Blastocystis* presence to certain bacterial microbiome compositions may be partly attributed to its anaerobic growth conditions [25, 40]. Recently, *Blastocystis* presence was associated to healthier diets and beneficial cardiometabolic outcomes in a large metagenomic study [41]. As a result of these latest findings, it has been argued that *Blastocystis* spp. may act more as an enabling factor in the gut microbiota than as an harmful intruder.

The potential occurrence of symptoms associated with *Blastocystis* spp. infection might be related to the distinction between carriership and actual colonization. The observed acquisition and disappearance of *Blastocystis* encountered in this case series could reflect the natural course of carriership. Multiple studies show that around 30% of *Blastocystis* infections clear spontaneously within a few weeks [27, 42, 43], while cases of persistent colonization lasting up to 10 years have also been reported [39]. Lastly, pathogenic potential and symptomatology may be related to the subtype [44] and/or load of these protozoa, which warrants further investigation.

The primary goal of (feces) donor screening is to minimize the risk of transferring pathogenic microbes. However, risk-benefit analysis and the cost-effectiveness of screening criteria should be evaluated and adjusted where needed. International guidelines for FMT donor screening differ in their recommendations to screen for *Blastocystis* spp. and exclusion of donors when present [14, 21, 45]. This variety is reflected in clinical practice, where there is a wide range of approaches for testing and reporting on *(Blastocystis)* screening in stool donors [46]. A recent systematic review evaluating 33 screening protocols worldwide proposed a new minimum set of recommended tests for stool donors, that did not include screening for *Blastocystis* spp [47]. As research on FMT continues to evolve, the criteria for selecting safe and effective feces donors must be updated accordingly. Our case series indicates that human transmission of *Blastocystis* ST3 by fresh FMT can occur, but this was only transient and without effect on clinical safety. Given the potential benefits associated with the presence of *Blastocystis* spp. in the gut microbiota, these results support the abandonment of *Blastocystis* spp. screening for feces donors, although larger studies are needed to verify these results.

## Electronic supplementary material

Below is the link to the electronic supplementary material.

Supplementary Material 1

## Data Availability

No datasets were generated or analysed during the current study.
