# Supplementary material for: Transient colonization with Blastocystis spp. after transmission via fecal microbiota transplantation
Source: Eur J Clin Microbiol Infect Dis. 2025 Apr 21;44(7):1621–7. doi: 10.1007/s10096-025-05124-6 (PMC12241144; doi:10.1007/s10096-025-05124-6)
Supplement: Supplementary file 1 — Supplementary Material 1 [file 10096_2025_5124_MOESM1_ESM.docx]

|  | **Screening** | **First FMT** | **Second FMT** |  |  |  |
| --- | --- | --- | --- | --- | --- | --- |
| **Timepoint (weeks)** | **-X** | **0** | **6** | **12** | **24** | **48** |
| **Patient:** |  |  |  |  |  |  |
| *Blastocystis* PCR and subtyping (Sanger)  - frozen sample (morning stool) | X | X | X | X | X | X |
| *Blastocystis* PCR and microscopy  - fresh sample | X |  | (X) | (X) |  |  |
| **Donor:** |  |  |  |  |  |  |
| *Blastocystis* PCR and subtyping (Sanger)  - frozen sample (morning stool) | X | X | X |  |  |  |
| *Blastocystis* PCR and microscopy  - fresh sample | X | (X) | (X) |  |  |  |

**Supplementary Table 1: Overview of sampling and performed tests for *Blastocystis* spp. identification in patients and feces donor.**

Abbreviations: FMT, fecal microbiota transplantation; PCR, polymerase chain reaction.

(X): not included in the FAIS study protocol; a fresh DFT was only performed for recipients 1 and 2 at week 6, and for recipient 1 at week 12.

|  |  |  | *Fresh feces sample* | | *Time (days)** | *Frozen feces sample* | |
| --- | --- | --- | --- | --- | --- | --- | --- |
|  | **Participant** | **Disease** | **DFT result** | **Microscopy evaluation** |  | **Blasto qPCR Cp** | **Sanger PCR result** |
| 1 | **FAIS Patient** | IBS | Positive | Unknown | 0 | 18,74 | Subtype 4 |
| 2 | **Donor** | HV | Positive | Many | 0 | 23,29 | Subtype 3 |
| 3 | **Donor** | HV | Positive | Many | 0 | 28,24 | Subtype 3 |
| 4 | **Donor** | HV | Positive | Many | 0 | 31,2 | Sacharomyces |
| 5 | **FAIS Patient** | IBS | Positive | Some | 1 | 29,88 | Subtype 2 |
| 6 | **FAIS Patient** | IBS | Positive | Some | 0 | 24,71 | Subtype 1 |
| 7 | **TURN2 Patient** | UC | Positive | Few | 0 | 28,48 | Subtype 6 ? |
| 8 | **Donor** | HV | Positive | Many | 0 | 16,15 | Subtype 3 |
| 9 | **TURN2 Patient** | UC | Positive | Many | 11 | 16,32 | Subtype 6 |
| 10 | **TURN2 Patient** | UC | Positive | Some | 0 | 18,02 | Subtype 3 |
| 11 | **TURN2 Patient** | UC | Positive | Some | -36 | Negative |  |
| 12 | **Donor** | HV | Positive | Moderate | 0 | 15,98 | Subtype 1 |
| 1 | **FAIS Patient** | IBS | Negative |  | 0 | Negative |  |
| 2 | **FAIS Patient** | IBS | Negative | - | 0 | Negative |  |
| 3 | **FAIS Patient** | IBS | Negative | - | 0 | Negative |  |
| 4 | **FAIS Patient** | IBS | Negative | - | 1 | Negative |  |
| 5 | **FAIS Patient** | IBS | Negative | - | 6 | Negative |  |
| 6 | **FAIS Patient** | IBS | Negative | - | 0 | Negative |  |
| 7 | **FAIS Patient** | IBS | Negative | - | 0 | Negative |  |
| 8 | **FAIS Patient** | IBS | Negative | - | 0 | Negative |  |
| 9 | **FAIS Patient** | IBS | Negative | - | 0 | Negative |  |
| 10 | **FAIS Patient** | IBS | Negative | - | -1 | Negative |  |

**Supplementary Table 2: Results from validation of used PCR for *Blastocystis* spp. determination on deep frozen samples, compared with previously determined *Blastocystis* status from fresh samples from the same individuals.**

Two *Blastocystis*-positive samples, initially detected in fresh feces, tested negative in their corresponding frozen samples; sample 4 had the lowest load (Cp = 31) and sample 11 had a lengthy gap of over a month between the sampling dates. *Days between fresh feces sample delivered (DFT performed) and frozen feces sample delivered. Abbreviations: IBS, irritable bowel syndrome; HV, healthy volunteer; UC, ulcerative colitis.

| Blas-F CGTTGTTGCAGTTAAAAAGCTCGT |
| --- |
| Blas-R GATTAATGAAAACATCCTTGGTAAATGC |
| Blas-P* CAgTTgggggTA+T+TCA+TA+T+TC |
| Blasto_seq_F TTgTTgCAgTTAAAAAgCTCgTAgTTgA |
| Blasto_seq_R CgCACTTgTTCATCTTCCATAAATC |

**Supplementary Table 3:** **Primers and probed that were used for the detection of *Blastocystis* spp.**

*Locked nucleic acid (LNA probe), + denotes LNA modification

|  | Feb 2019 | March 2019 | Dec 2019 | Feb 2020 | Feb 2020 | June 2020 | Sept 2020 | Jan 2021 | Feb 2021 |
| --- | --- | --- | --- | --- | --- | --- | --- | --- | --- |
| PCR | Positive | Positive | Positive | Positive | Positive | Positive | Positve | Positive | Positive |
| Microscopic amount | A few | Some | Some | Many | Many | Sporadic | Many | Many | Many |
| Co-occurrence protozoa | *E. coli* | *E. coli*  *E. nana* cysts | *E. coli*  *E. nana* cysts | *E. coli*  *E. nana* cysts | *E. coli* | *None* | *E. coli*  *E. nana* cysts | *E. coli*  *E. nana* cysts  *E. histolytica/dispar* cysts  *E. hartmanni* cysts | *E. coli*  *E. nana* cysts |

**Supplementary table 4: Timeline of *Blastocystis* carriership with microscopic quantification and co-occurrence of apathogenic protozoa in feces donor over 2 years.**
